# City and County of San Francisco

**Published:** 1916-04

**Authors:** Wm. C. Hassler

**Affiliations:** Health Officer


### City and County of San Francisco.

Department of Public Health replied as follows:

Relative to your query of recent date, submit the following:—

1. In what diseases is dismissal from quarantine based on negative bacteriologic evidence?

A. Diphtheria and Typhoid Fever. (Specific vaginitis and ophthalmia neonatorum when in city hospitals).

2. In what diseases is dismissal from quarantine based on an arbitrary time limit?

A. Scarlet Fever and Poliomyelitis.

3. Is there any evidence that the latter method is not efficient or, on the other hand, that quarantine is unnecessarily prolonged?

A. No, excepting in Poliomyelitis, where the quarantine by rule is fixed at three months, which rule might be modified, as even up to the present no successful method, even inoculating monkeys with washings of nose and throat, has proven satisfactory.

4. Is the former method applicable to typhoid?

A. Bacteriological examination of stools are made of all typhoid fever cases in this city before they are released. Two negatives with five days interval is the rule. Carriers of diphtheria are treated as cases of diphtheria, the placard differing only in the word "carrier" being added.

Respectfully,

WM. C. HASSLER, Health Officer.

